# Trends of HIV/AIDS Phenomenon Dynamics in Romania from 2017–2027

**Published:** 2019-10

**Authors:** Andrioni Felicia

**Affiliations:** Department of Socio-Human Sciences, Faculty of Sciences, University of Petroşani, Petroşani, Romania

**Keywords:** HIV/AIDS phenomenon, Dynamic, Trends, Romania

## Abstract

**Background:**

HIV infection remains of major public health importance in all world and in Romania. In Romania there are a large number of long-term survivors coming from the 1987–1990 generation, the circumstances are due to the fact that an increased number of HIV-infected persons receive a specific therapy.

**Methods:**

The study was conducted using sociological analysis methods such as comparative analysis, statistical analysis, and with the help of the method of regression analysis, to capture the dynamics of the HIV / AIDS phenomenon in Romania, as well as forecasting the dynamics of the phenomenon over the next decade.

**Results:**

This longitudinal analysis of the statistical data provided by the Matei Bals Institute of Romania during the period 2004–2016, shows a progressive increase 1.4 times higher in 2016 compared to 2004, the prediction of the extension of the HIV / AIDS phenomenon for the period 2017–2027, in Romania being almost constant. Regarding the prognosis of the number of persons affected by HIV / AIDS in Romania, for the next 10 years an upward dynamic is predicted with an increasing annual rate of 506 new persons taken into evidence. As the prediction function shows, the trend the access to treatment is increasing for the next decade. After 2006 in Romania, the deaths dynamics is an exponential decreasing one, keeping the same rate of decrease, until 2015.

**Conclusion:**

The HIV/AIDS pandemic has changed considerably in the past 27 years. AIDS incidence and mortality in industrialised countries have fallen, and paediatric HIV disease has almost been eliminated as a public health issue, largely through antiretroviral drugs.

## Introduction

In Europe, Romania detained until recently among the first place in terms of the number of AIDS-infected children, while, in terms of the total number of cases, it was closer to the Western countries than to the countries in Central and Eastern Europe, during the period 1987–1990 (1). In Romania, the highest rate of AIDS infection in children was reported at the end of 1989, while 90% of the cases were registered at the beginning of the 1990s. During the same period, the rate of HIV infection in adult population was smaller, although it subsequently manifested an increase, beginning with 2007 (1).

The HIV / AIDS phenomenon in Romania has as a distinctive feature the “presence of a relatively compact group of young people infected by nosocomial channels in childhood through the use of non-sterile medical equipment and products untested for HIV (2). In the early 2000s, Romania had more than half of the cases of Pediatric HIV / AIDS in Europe, a situation resulting from the transmission of the infection in the Romanian health system (3), and it is estimated that over 10,000 children were infected with HIV, of whom 3,000 died, the other 7,000 being long-term survivors with the current age range between 26–30 years (4).

Nowadays, Romania registers an increased number of long-time survivors belonging to the 20–29 age group, with origins in the 1987–1990 group of infected individuals; the circumstances are due to the fact that an increased number of HIV-infected persons receive a specific therapy in comparison with the total number of infected persons. On 30^th^ of June 2016 in Romania, there were 21,702 HIV-infected or AIDS-suffering people alive (5).

The UNAIDS targets globally and implicitly for Romania are that by 2020, 90% of the persons are tested for HIV, 90% of the patients diagnosed to have access to treatment, 90% of the HIV-positive people to have an undetectable viral load.

This study was conducted in a national context aimed at identifying the national HIV / AIDS phenomenon on the one hand, and the prognosis of the HIV / AIDS phenomenon in the next 10 years on the national level.

## Materials and Methods

The research methods of the analysis were document analysis, comparative analysis, statistical analysis and simple regression analysis of linear type, power type, exponential and logarithmic.

The proposed model for HIV / AIDS prognosis nationally was based on specific calculation formulas that lead to a prognosis in time of the phenomenon.

In order to establish the relevant indicators in the phenomenon analysis we studied the literature and other analyses conducted by experts. In order to accurately reflect the HIV / AIDS phenomenon in Romania, we took into consideration certain analysis parameters already used by the Regional Centers for Evaluation and Monitoring of HIV infection / AIDS disease and processed at the Department for Monitoring and Evaluation of HIV / AIDS Infection in Romania at the National Institute of Infectious Diseases “Prof.dr.Matei Balş” – Bucharest (6).

The statistical data provided by the Matei Bals Institute of Romania is public, the statistical data were used only for research purpose, and there were not ethical issues in this study.

Depending on the parameters analysed, the following analysis indicators were structured for the analysis of HIV / AIDS dynamics in Romania and the prognosis of the phenomenon over the next 10 years: I1 Total HIV / AIDS (cumulative) in Romania; I2 Total AIDS deaths (cumulative) in Romania; I3 Total HIV + AIDS lost from evidence; I4 Total HIV + AIDS patients in active evidence.

## Results

Analysis of the dynamics of the HIV / AIDS phenomenon in Romania and the prognosis of this phenomenon in the period 2017–2027

In the following discussion, we highlight the HIV / AIDS depending on each distinct in Romania:

### HIV/AIDS (cumulative) in Romania

**Fig. 1:**
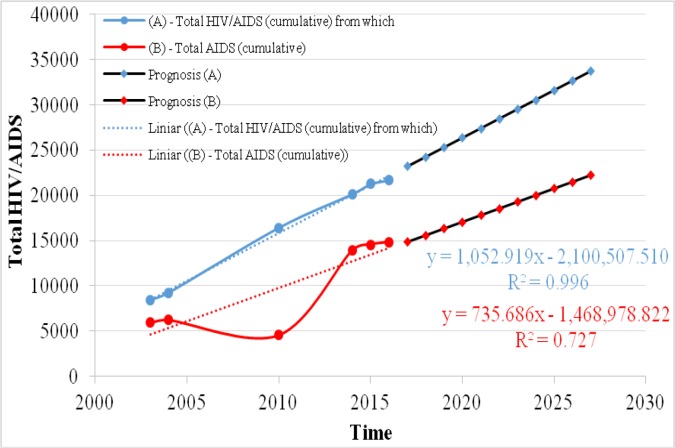
Prognosis of total HIV/AIDS (cumulative) in Romania (number)

From the longitudinal analysis of the data provided by the Matei Bals Institute of Romania, regarding the situation in Romania, for the period 2004–2016 there is a progressive increase 1.4 times higher in 2016 (21702 persons) compared to 2004 (15471 persons ). The prediction of the spread of the HIV / AIDS phenomenon for the period 2017–2027 is almost constant, as it results from the analysis of the linear regression of the data shown in Fig. 1, resulting in an increase of 1.45 times the next 10 years, from 23,230 in 2017 to 33759 in 2027, with an approximation of 72% (with a coefficient of determination R^2^=0,727).

**Fig. 2:**
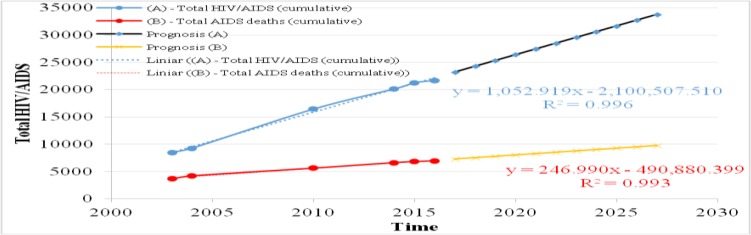
Prognosis of total AIDS deaths (cumulative) in Romania (number)

From the official data of the National Board of Fight Against AIDS, “Prof. Dr. Matei Balş” Institute of Infectious Diseases we can see in Fig. 1 that, as regards the number of persons affected by AIDS in Romania there is an increase of 60.32% in the number of people affected by AIDS in the period 2004–2016 (from 9258 in 2004 to 14835 in 2016), the increase in this number being explained in the natural context of the evolution of HIV disease and the onset of AIDS disease. However, in Romania, due to the universal access to medication, treatment and care for people affected by AIDS, there is a slight decrease in the number of deaths of persons affected by AIDS (Fig.2).

We find that the share of total cumulative HIV / AIDS in relation to the number of AIDS patients is relatively constant at a rate of 1.67 in 2004, 1.38 in 2010 and 1.46 in 2016 respectively.

With regard to the prognosis of AIDS dynamics over the next decade, with the help of the linear regression analysis one foresees a 1.49-fold increase (from 14,900 in 2017 to 22,257 in 2027) with a similar growth rate to the HIV / AIDS phenomenon cumulatively predicted over the same period.

At the end of 2016, as a result of the analysis of the data recorded in the Matei Bals Institute's national database - over the past 25 years, respectively 21702 cumulated cases, Romania continues to have a large number of “long-term survivors” aged between 20–24 years, 46% being women.

### AIDS deaths (cumulative) Romania

In terms of predicting the dynamics of HIV / AIDS deaths over the next decade (Fig. 2), the linear regression analysis predicts a death rate 1.33 times higher in 2027 compared to 2017 (from 7298.4 in 2017 to 9768.3 in 2027, with a very good determination coefficient R^2^=0,993).

### HIV+AIDS lost from evidence in Romania

In Romania, the monitoring of people affected by HIV / AIDS is extremely important, especially as there are often situations when, despite the fact that a significant number of cases registered in the national surveillance network are highlighted, these cases are subsequently lost from the evidence for more or less objective reasons.

**Fig. 3:**
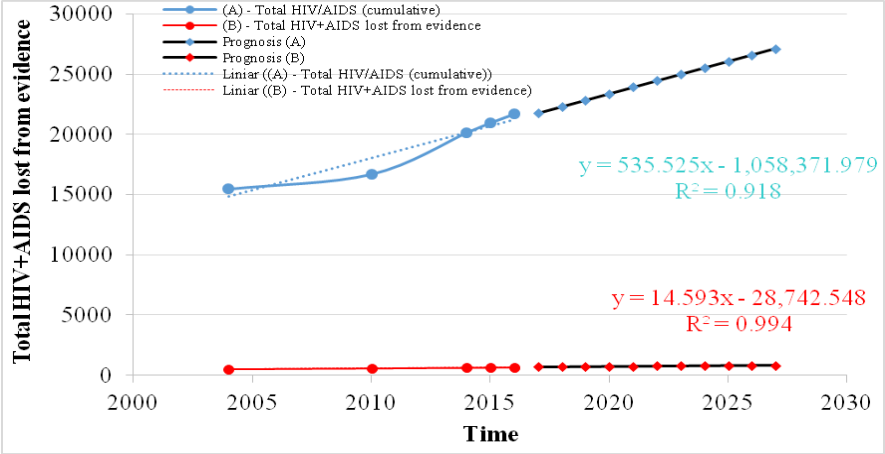
Prognosis of total HIV+AIDS lost from evidence

It is certain that those cases whose “trace” has been lost can no longer have access to investigation, medication and treatment, and the progression of their disease cannot be known. For this reason, one of the important national indicators highlighted in official statistics refers to the number of people affected by HIV / AIDS lost from records. From the statistical analysis of the official data provided by the Matei Balş Institute it is found that in 2004, 3.26% of the total number of registered persons were lost from the records, there being a constant rate of these losses of 3.50% in 2010, respectively 3, 15% in 2016 (Fig.3).

### HIV+AIDS patients in active evidence in Romania

In order to provide treatment and care for HIV / AIDS, Romania has a well-organised system at present.

Medical advances over the past 15 years in the field of HIV / AIDS and the introduction of appropriate antiretroviral therapies have enabled a considerable number of affected people from 1987–1990 to survive and reach maturity. Most HIV-infected patients are between the ages of 26–30. Pediatric AIDS cases are currently low due to a number of factors.

**Fig. 4:**
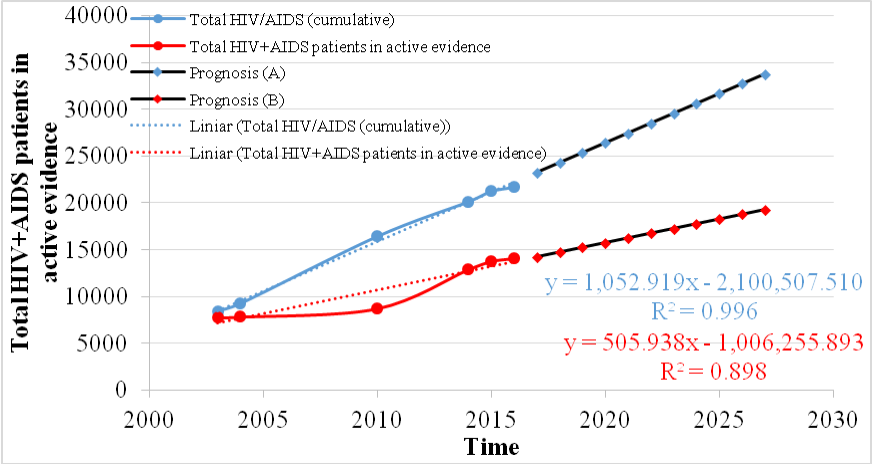
Prognosis of total HIV+AIDS patients in active evidence between 2017 and 2027

Based on the longitudinal analysis of statistical data from Matei Balş Institute there is an increasing dynamics of the number of persons with active evidence of HIV / AIDS in the period 2004–2016, registering a gradual increase 1.55 times higher (Fig.4) in 2016 (12,196 persons) compared to 2004 (7854 persons), due to some more strategic directions for surveillance, control and prevention of cases of HIV / AIDS infection that were finalized and applied in 2004–2007. Following the analysis of the linear regression, regarding the prognosis of the number of people affected by HIV / AIDS that will be taken into evidence, an upward dynamic is predicted for the next 10 years with an increasing annual rate of 506 newly registered persons, from 14221/2017 to 19280/2027 (with a very good determination coefficient R^2^=0,898).

## Discussion

The HIV/AIDS pandemic has tremendously spread at a global level with serious implications upon population (7).

One of the Central and Southeast European countries with a significant number of HIV-AIDS cases and with the highest survival rate in Central and Eastern Europe is Romania. More than 50% of the cases detected in the 1990s are alive due to access to modern treatment schemes, since the life expectancy was only three months in the 1990s (8). For this reason, it is explained that the life expectancy of people affected by HIV / AIDS has increased and the number of AIDS-related deaths has been kept under control, registering a 60% increase in 2016 compared to 2004 (from 4231 deaths Registered in 2004 and 6940 respectively in 2016), even though the spreading of the HIV / AIDS phenomenon in the same period increased by 71% in 2016 compared to 2004.

The emergence and spread of HIV infection in Romania had as its main causes the poor endowments in the hospitals with poor sanitary instruments in the period before 1989 (December 1989 post-communist revolution in Romania), which generated the inappropriate and repeated use of syringes and unsterilized needles in the communities and medical centres, due to transfusions with infected blood associated with the lack of reagents needed to HIV screening diagnosis in blood collection centres due to socioeconomic factors (9). On the background of the deficient medical instruments from the period before 1989, we find that the highest number of HIV-infected children in Europe, Romania had 60% of the total paediatric AIDS in Europe due to the parenteral transmission of HIV.

In June 2016 the HIV/AIDS global community aimed to reduce new infections to less than 500,000 by 2020 (10) and at the meeting in September 2015 the global community committed to ending the AIDS epidemic by 2030 (11). This study confirmed some aspects mentioned before, and predicted that in the next decade, the new HIV infections keeping a decreasing trend and the dynamics of AIDS –related mortality after 2006, are decreasing exponentially. “Across this time frame, the AIDS response has grown in maturity and sophistication, built on robust collaboration and singular purpose” (12).

Regarding the situation in Romania, there is a possibility that the phenomenon on the national level is far from being correctly estimated. There are new cases that are discovered every day, but for reasons related to confidentiality or because of the refusal of patients to be taken into the evidence of specialised compartments in the infection treatment, there are difficulties in closely monitoring new cases.

In the current context of the European Union, the evolution of the HIV-AIDS phenomenon in Romania was a carefully controlled phenomenon, especially after 2000, when the National HIV-AIDS Strategy was launched for the next period. By preventing new HIV infections, at the national level, morbidity and mortality due to human immunodeficiency virus (HIV) infections can be reduced through antiretroviral treatment interventions.

## Conclusion

In Romania, the longitudinal analysis of the statistical data provided by Matei Bals Institute for the period 2004–2016 shows a progressive increase 1.4 times higher in 2016 compared to 2004, the prediction of the extension of the HIV / AIDS phenomenon for the period 2017–2027 being almost constant, as is shown by the linear regression analysis of the data, resulting in a 1.45-fold increase over the next 10 years. Likewise, this prognosis is worrying and warns about the imminence of the phenomenon spread on national level in Romania over the next 10 years.

A favourable aspect in Romania is the access to treatment with an increasing trend from 2000 to 2015. As the prediction function shows, the trend is increasing for the next decade, which means there is hope that in 10 years, the increase in access to treatment shall be more than 10 times higher.

## Ethical considerations

Ethical issues (Including plagiarism, informed consent, misconduct, data fabrication and/or falsification, double publication and/or submission, redundancy, etc.) have been completely observed by the authors.
